# Study of Posterior Cerebral Artery in Human Cadaveric Brain

**DOI:** 10.1155/2015/681903

**Published:** 2015-08-24

**Authors:** S. A. Gunnal, M. S. Farooqui, R. N. Wabale

**Affiliations:** Department of Anatomy, Rural Medical College, Post. Loni, Taluka Rahata, Ahmednagar District, Maharashtra 413736, India

## Abstract

*Objective*. Basilar artery (BA) terminates in right and left posterior cerebral arteries (PCAs). Each PCA supplies respective occipital lobe of the cerebrum. The present study is designed to know the morphology, morphometry, branching pattern, and symmetry of PCA. *Methods*. The study included 340 PCAs dissected from 170 human cadaveric brains. *Results*. Morphological variations of P1 segment included, aplasia (2.35%), hypoplasia (5.29%), duplication (2.35%), fenestration (1.17%), and common trunk shared with SCA (1.76%). Morphological variations of origin of P2 segment included direct origin of it from BA (1.17%) and ICA (2.35%). Unusually, two P2 segments, each arising separately from BA and ICA, were observed in 1.17%. Unilateral two P2 segments from CW were found in 0.58%. Morphological variations of course of P2 were duplication (0.58%), fenestration (0.58%), and aneurysm (1.76%). Unilateral P2 either adult or fetal was seen in 4.71%. The group II branching pattern was found to be most common. Asymmetry of P2 was 40%. Morphometry of P2 revealed mean length of 52 mm and mean diameter of 2.7 mm. *Conclusion*. The present study provides the complete anatomical description of PCA regarding morphology, morphometry, symmetry, and its branching pattern. Awareness of these variations is likely to be useful in cerebrovascular procedures.

## 1. Introduction

Basilar artery (BA) terminates in right and left posterior cerebral arteries (PCAs) [1]. PCA runs laterally and parallel with the superior cerebellar artery and receives the posterior communicating artery (PCoA) in its course. The point of joining of PCoA to PCA divides PCA into two segments, proximal P1 and distal P2. Thus, P1 segment of PCA is from the basilar bifurcation to the junction with the PCoA while P2 segment is from the junction with the PCoA to the termination of PCA. Each segment gives off groups of central and cortical branches that supply distinct anatomic territories like brainstem, thalamus, and ventricles. Cortical branches from PCA are anterior temporal artery, posterior temporal artery, parahippocampal artery, medial occipitotemporal artery, lateral occipitotemporal artery, calcarine artery, and parietooccipital artery [2].

Few studies are solely devoted to the anatomy of PCA. The aim of the present work is to find out the morphological variations, branching pattern, symmetry, and morphometry of PCA.

## 2. Materials and Methods

The study was conducted in the Department of Anatomy, Rural Medical College, PIMS, Loni, after clearance from Institutional Ethical Committee (PIMS/PhD/RC/2013/28). Three hundred and forty PCAs from 170 formalin preserved brains of adult human cadavers, of unknown age and cause of mortality, were included in the study. All cadavers appeared to be adults with approximate age between 40 and 60 years. The brains with gross morphological abnormalities were excluded. Arachnoid mater over interpeduncular fossa was removed carefully and the circle of Willis (CW) was exposed. Basilar artery (BA) was identified and carefully dissected to get bilateral posterior cerebral arteries (PCAs). PCAs on either side were carefully dissected from their origin to their termination. All the branches arising from PCA were carefully dissected to get the better view of branching pattern. Variations of PCAs and branching pattern were noted and photographed. The arterial networks of the CW along with the termination of BA and PCA of both sides were delicately separated from brain tissue and intact entire dissected vasculature was removed and pasted on the black plastic sheets. The study of branching pattern of PCA was done as per the scheme described by Ladzinski and Maliszewski [3]. Dimensions of the PCA were measured using vernier caliper with the least count of 0.01 mm. Length and external diameters of P1 and P2 segments of PCA were measured. The percentage variation, mean of length, and external diameters of PCA were estimated. The length and external diameter of these two segments of PCA were measured. The length of P1 segment was taken from its origin up to the PCoA, while the length P2 segment was taken from the PCoA to the termination of the main trunk of PCA. External diameter was measured in the middle of the P1 and P2 segments.

## 3. Observations and Results

### 3.1. Morphology of PCA

The junction of posterior communicating artery (PCoA) with PCA divides the course of PCA into two parts. The morphology of prejunctional segment (P1) and the postjunctional segment (P2) is described separately.

### 3.2. Morphological Variations of P1

**Figure 1:**
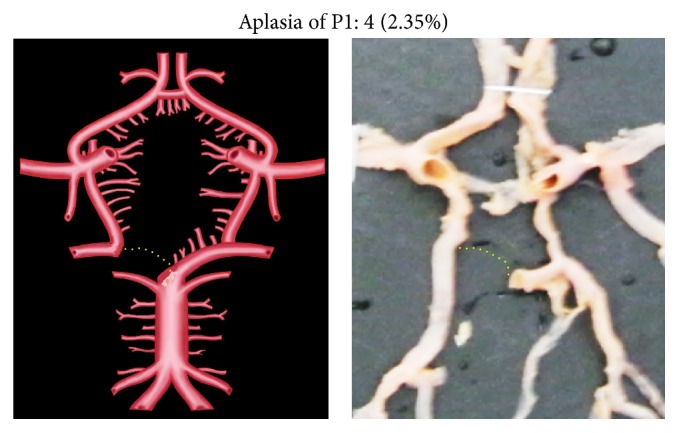
P1 aplasia. The dotted line shows the absent P1 segment.

**Figure 2 fig2:**
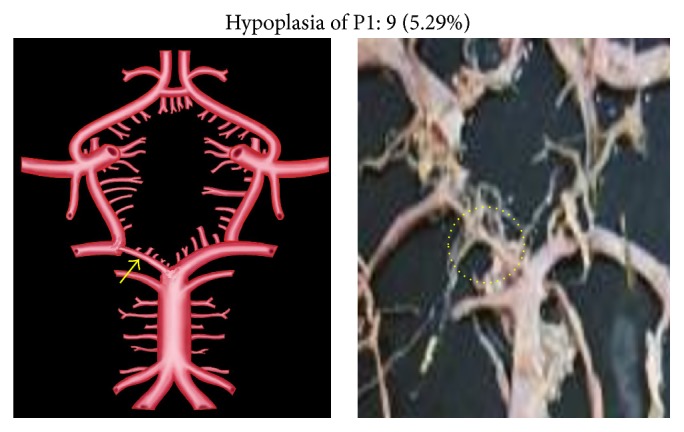
P1 hypoplasia. The arrow and dotted circle show the hypoplastic P1 segment.

**Figure 3 fig3:**
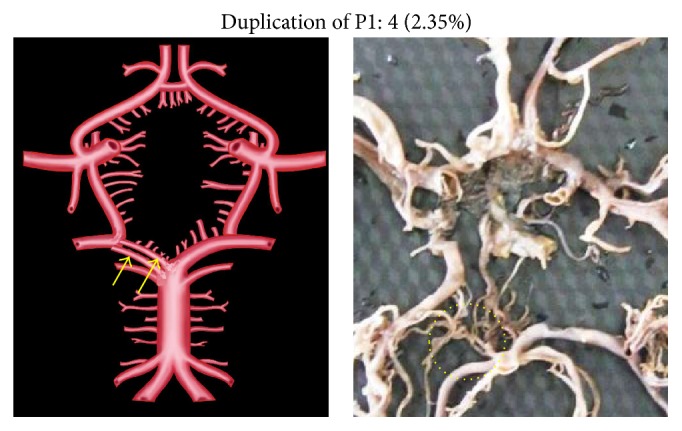
P1 duplication. Arrows and dotted circle show the duplicated P1 segment.

**Figure 4 fig4:**
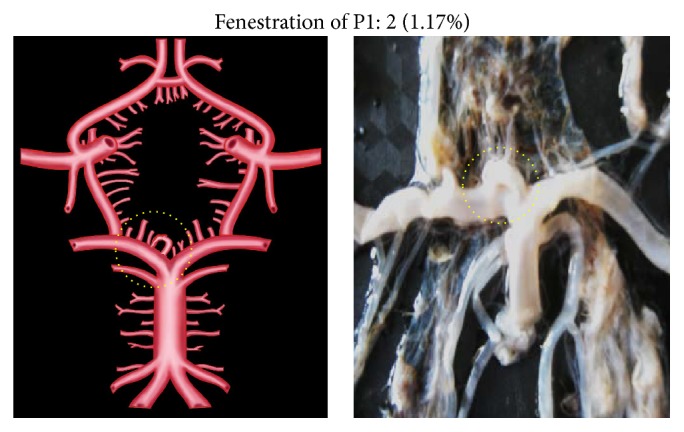
P1 fenestration. Dotted circle shows the fenestrated P1 segment.

**Figure 5:**
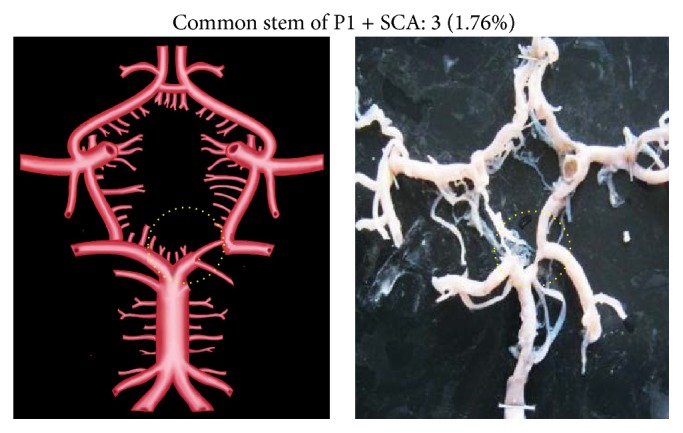
P1 and SCA common stem. Dotted circle shows the common stem for SCA and P1 segment.

Morphological variations of P1 in the present study included aplasia, hypoplasia, duplication, fenestration, and an unusual origin. The morphological variations of P1 were seen in 22 specimens (12.94%). Aplasia was seen in 4 specimens (2.35%), hypoplasia in 9 (5.29%), duplication in 4 (2.35%), fenestration in 2 (1.17%), and common stem of origin with SCA in 3 (1.76%). Variations of P1 described above, along with coupled schematic presentation, are shown in Figures 1 [Fig fig2] [Fig fig3] [Fig fig4]–5.

### 3.3. Morphological Variations of P2

**Figure 6:**
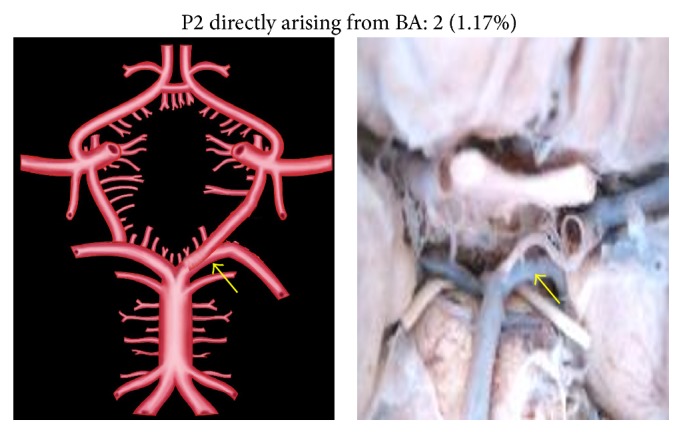
P2 directly arising from BA. The arrow shows the direct origin of P2 from BA.

**Figure 7:**
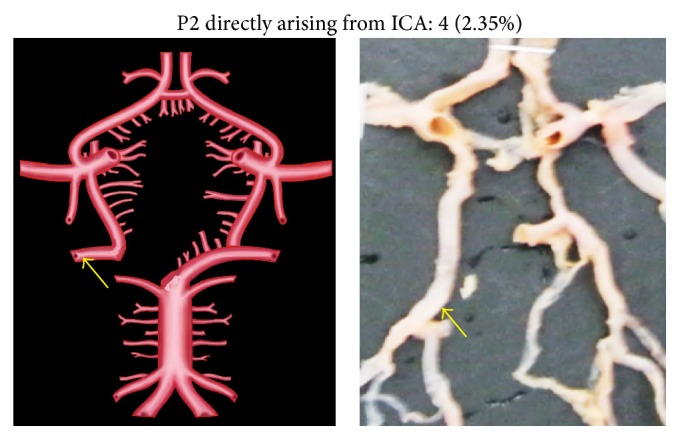
P2 directly arising from ICA. The arrow shows the direct origin of P2 from ICA.

**Figure 8:**
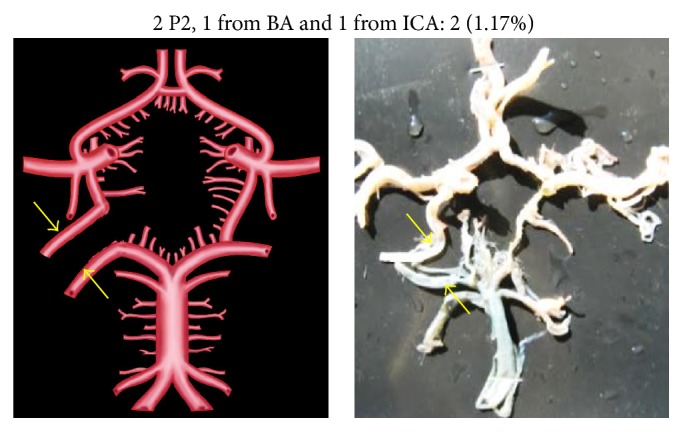
Two P2, one arising from BA and one from ICA. Two arrows show two P2, one originating from BA and one from ICA.

**Figure 9:**
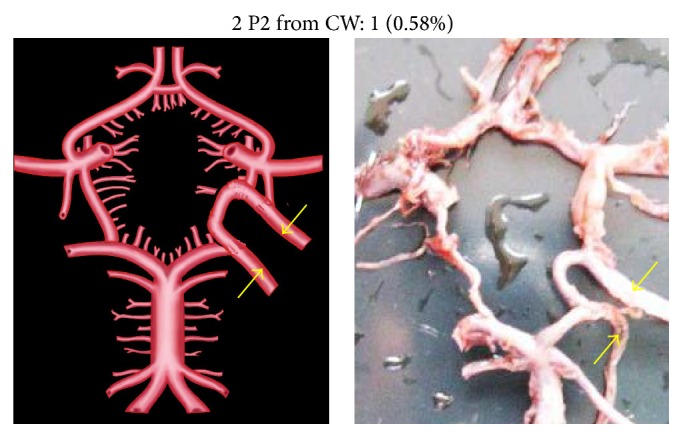
Two P2 both arising from extra loop in CW. Two arrows show two P2 originating from extra loop in CW.

An unusual variation of P2 segment in two specimens (1.17%) was its direct origin from basilar artery (BA) to which the respective PCoA joined at the point of bifurcation of BA (Figure 6). In four cases (2.35%), the P2 segment was directly arising from internal carotid artery (ICA) (Figure 7). This observation was coupled with aplastic P1 segment. Two individual P2 segments, one arising from BA and the other from ICA, were seen in 2 specimens (1.17%) (Figure 8). There was no communication between the PCoA and PCA. Two separate P2 segments, directly arising from extra loop of CW, were seen in 1 specimen (0.58%) (Figure 9).

### 3.4. Other Morphological Variations of P2

Duplication, fenestration, and associated aneurysm were the other morphological variations of P2 segment.

**Figure 10:**
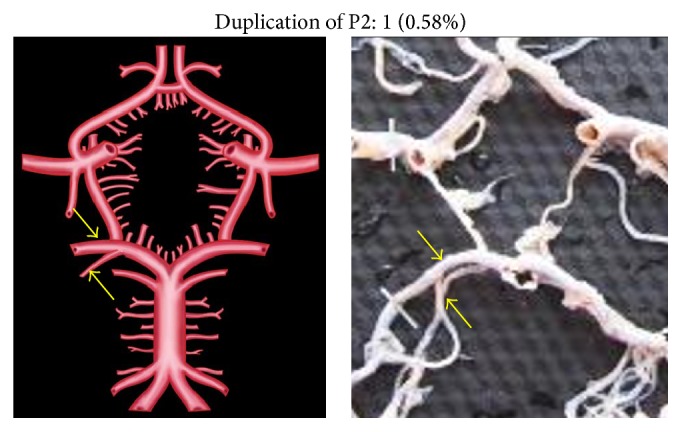
Duplication of P2. Two arrows show duplicated P2.

**Figure 11:**
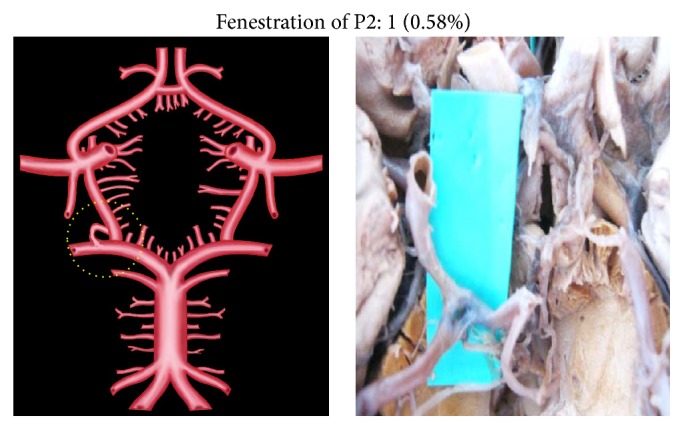
Fenestration of P2. Dotted circle shows fenestrated P2.

**Figure 12:**
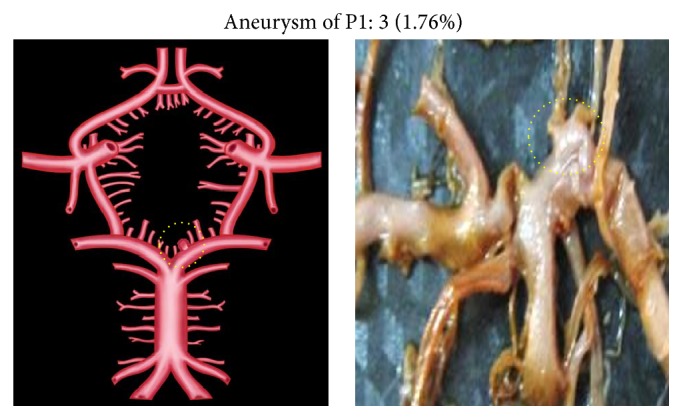
Aneurysm of P2. Dotted circle shows aneurysm of P2.

Unusually duplicated P2 segments and fenestrated P2 segment were noted, each in two individual specimens (0.58%) (Figures 10 and 11). Fenestration was present at the junction of PCoA, P1, and P2 (Figure 11). Aneurysm associated with P2 was seen in 3 specimens (1.76%) (Figure 12).

**Figure 13:**
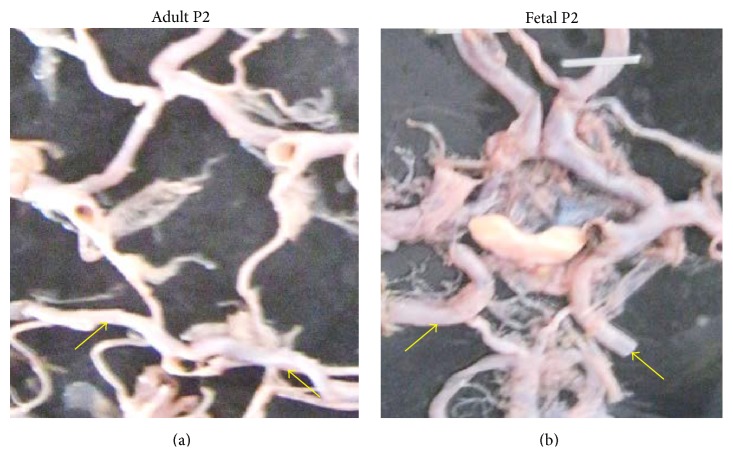
(a) Bilateral adult type of P2. (b) Bilateral fetal type of P2.

**Figure 14:**
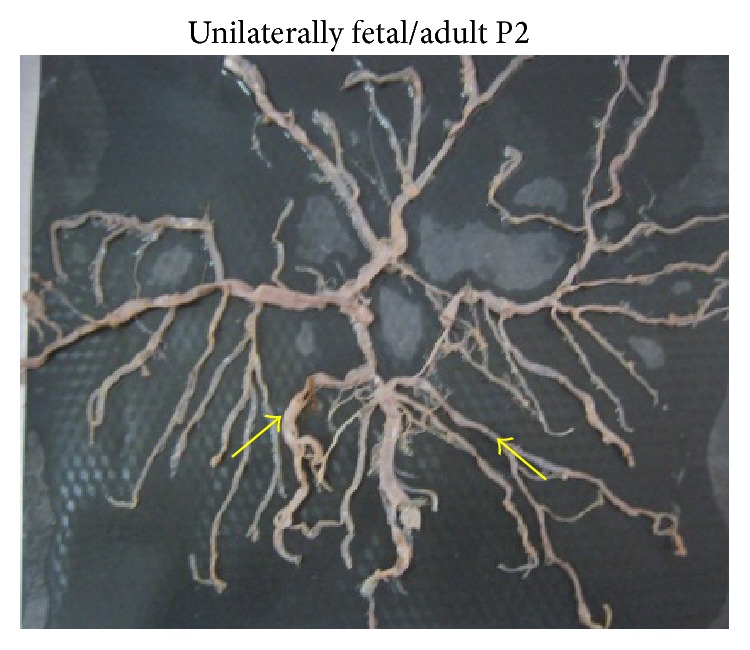
Bilateral adult/fetal type of P2.

Adult P2 segments and fetal P2 segments were bilaterally found in 142 (83.52%) and 20 (11.77%) brains, respectively (Figures 13(a) and 13(b)). Unilateral presence of either adult or fetal P2 segment was seen in 8 brains (4.71%) (Figure 14).

### 3.5. Branching Pattern of PCA

An unusual origin of superior cerebellar artery (SCA) from PCA was seen in 3 specimens (0.88%).

**Figure 15:**
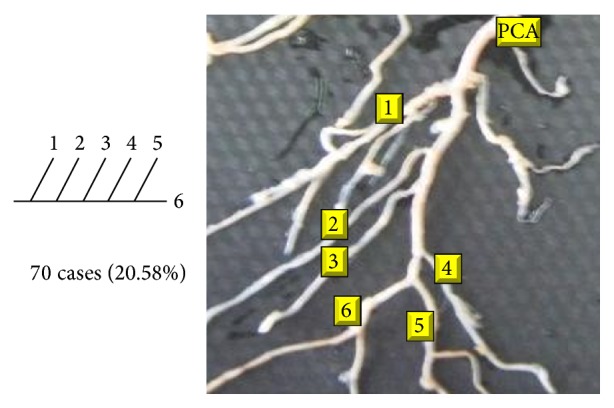
Type 1 branching pattern of PCA.

**Figure 16 fig16:**
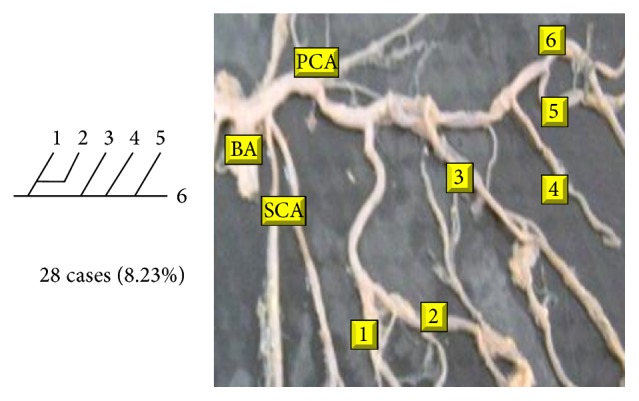
Type 2 branching pattern of PCA.

**Figure 17 fig17:**
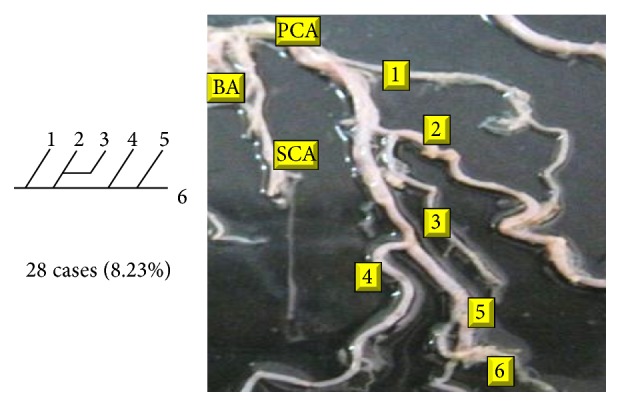
Type 3 branching pattern of PCA.

**Figure 18 fig18:**
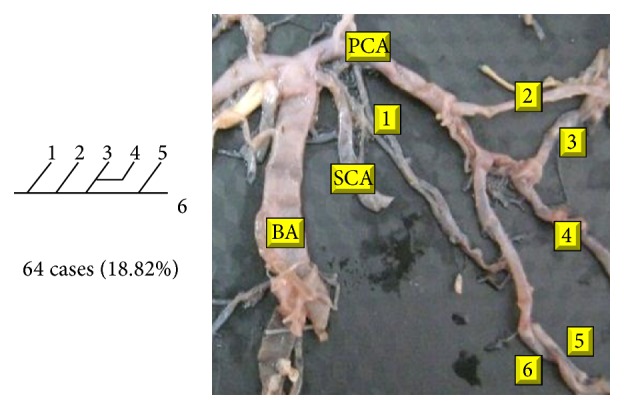
Type 4 branching pattern of PCA.

**Figure 19 fig19:**
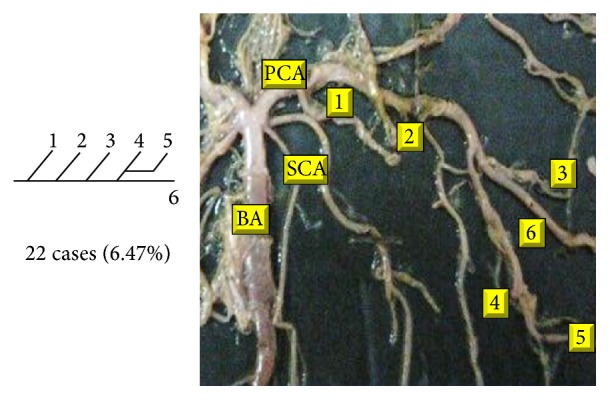
Type 5 branching pattern of PCA.

**Figure 20 fig20:**
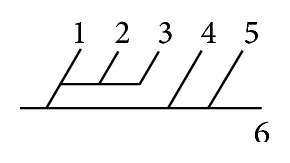
Type 6 branching pattern of PCA.

**Figure 21 fig21:**
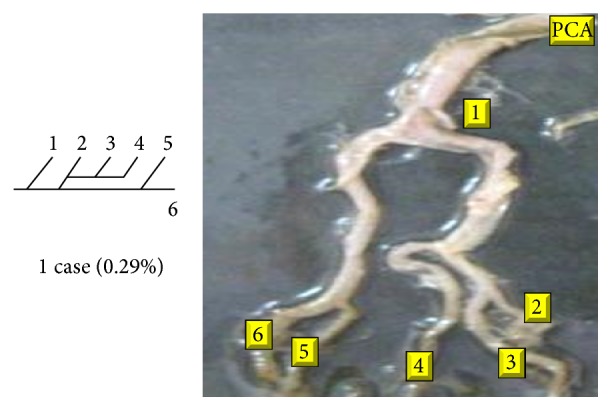
Type 7 branching pattern of PCA.

**Figure 22 fig22:**
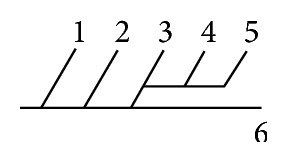
Type 8 branching pattern of PCA.

**Figure 23 fig23:**
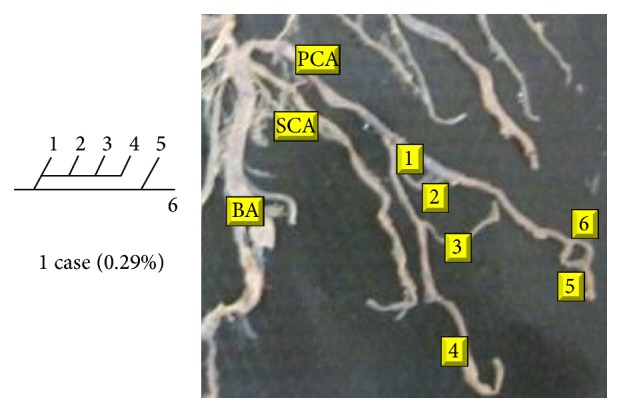
Type 9 branching pattern of PCA.

**Figure 24 fig24:**
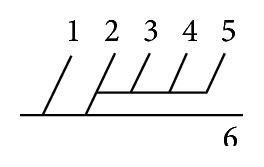
Type 10 branching pattern of PCA.

**Figure 25 fig25:**
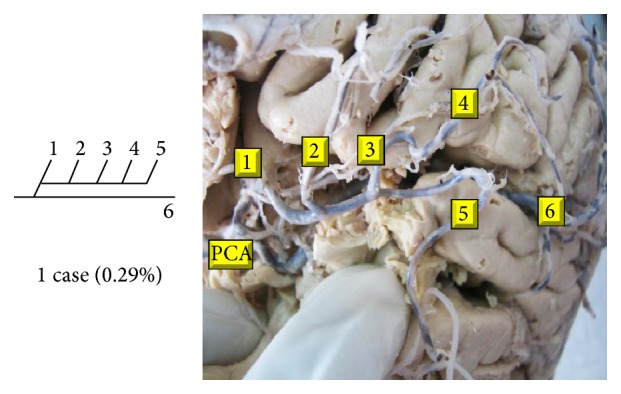
Type 11 branching pattern of PCA.

**Figure 26 fig26:**
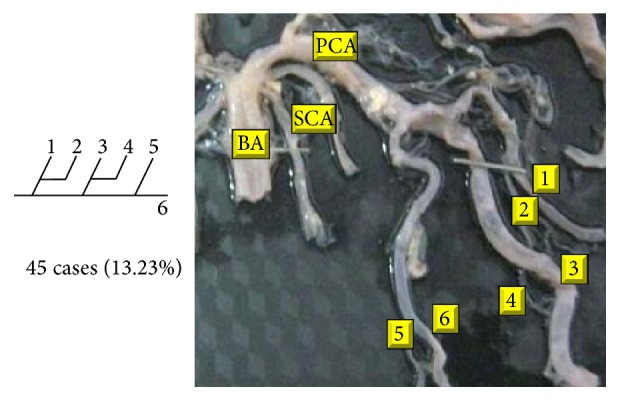
Type 12 branching pattern of PCA.

**Figure 27 fig27:**
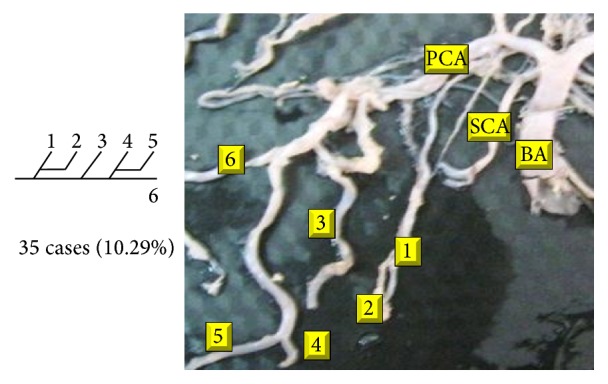
Type 13 branching pattern of PCA.

**Figure 28 fig28:**
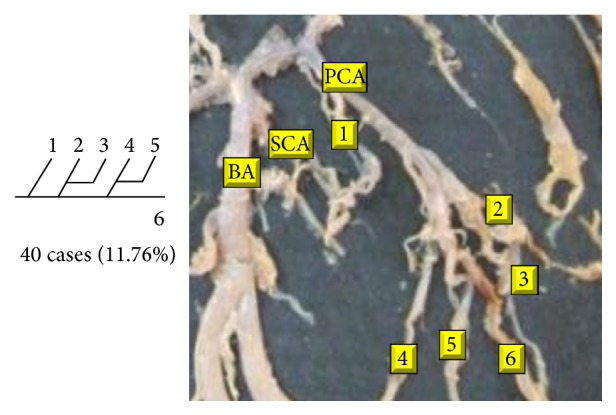
Type 14 branching pattern of PCA.

**Figure 29 fig29:**
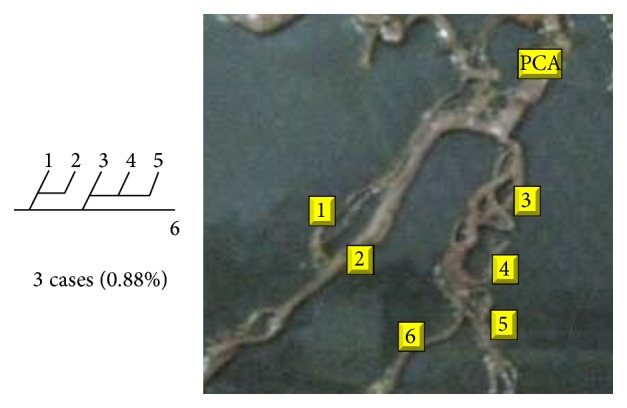
Type 15 branching pattern of PCA.

**Figure 30:**
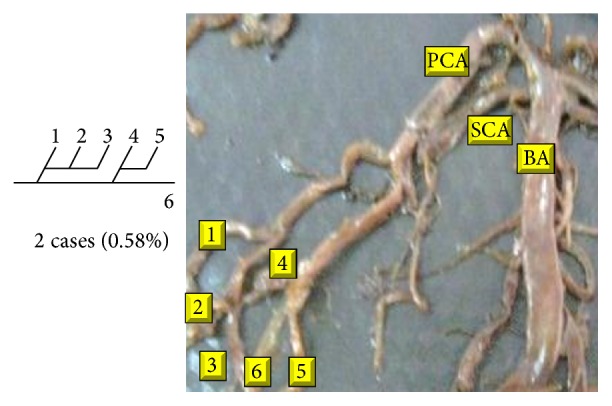
Type 16 branching pattern of PCA.

Of 16 different possibilities of the branching patterns, described by Ladzinski and Maliszewski [3], we could find 13 types only. The branching pattern type number 1 of Group I was the most common of all and was observed in 70 specimens (20.58%). The percentage of branching pattern of Group III, Group IV, and Group V was 0.29% and was the least of all, represented by a single observation per group. The rest of the branching patterns observed are narrated in Table 1. Photographs of different branching patterns observed are shown in Figures 15 [Fig fig16] [Fig fig17] [Fig fig18] [Fig fig19] [Fig fig20] [Fig fig21] [Fig fig22] [Fig fig23] [Fig fig24] [Fig fig25] [Fig fig26] [Fig fig27] [Fig fig28] [Fig fig29] –30.

### 3.6. Symmetry of PCA

**Figure 31:**
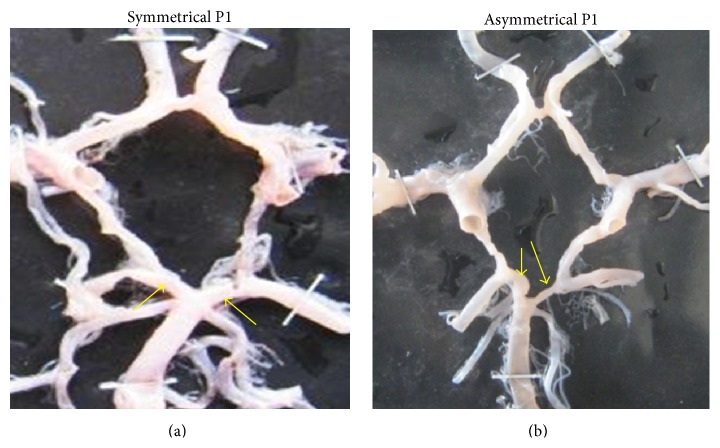
(a) Symmetry of P1; (b) asymmetry of P1.

**Figure 32:**
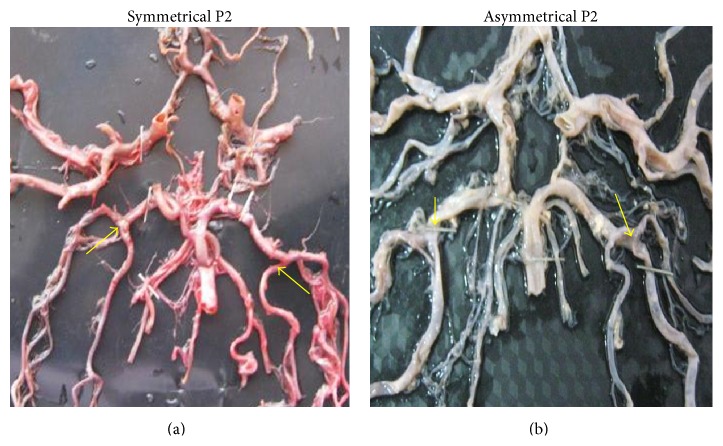
(a) Symmetry of P2; (b) asymmetry of P2.

Symmetry of P1 was observed in 119 specimens (70%) while asymmetry was seen in 51 specimens (30%) (Figure 31). Symmetry of P2 was observed in 102 specimens (60%) and asymmetry was seen in 68 specimens (40%) (Figure 32). Asymmetry was mostly associated with its branching pattern.

### 3.7. Morphometry

Morphometric details of P1 and P2 segments are given in Table 2.

## 4. Discussion

The present study was especially planned to know the extent of variations in morphology and morphometry of PCA. The course of PCA is divided in P1 and P2 segments by the joining of PCoA. Findings of morphology of P1 of the present study, along with the findings by other authors, are provided in Table 3 for comparison.

Since P1 segment of PCA forms the boundary of CW, it has attracted attention of many research workers. In comparison with P1, reports of P2 are limited. The present study also describes the extent of morphological variations, morphometry, symmetry, and branching pattern of P2 segment of PCA. Unlike other research workers who have studied the adult and/or fetal configuration of P2 segment unilaterally, the present study considered the adult and/or fetal configuration of P2 segment bilaterally in individual brain specimens together. Bilateral adult pattern of P2 segment was seen in 83.52% while bilateral fetal pattern was seen in 11.77%. The unique finding of the present study is that the presence of unilateral adult configuration with contralateral fetal configuration of P2 segment of PCA was 4.71%. We could not find any study reporting the presence of unilateral adult configuration with contralateral fetal configuration of P2 segment of PCA.

In normal circumstances, occipital lobes of cerebrum enjoy nutrition from both sources contributing to the CW, that is, ICA and vertebrobasilar artery. Presence of morphological variation usually produces hemodynamic changes in respective regions of the body. Presence of morphological variations of P2 segments of PCA is likely to change hemodynamic status of occipital lobe. So far, there are no reports directed to studying such hemodynamic changes due to morphological variations of P1 and P2 segments of PCA. Significance of this likely hemodynamic change because of anatomic anomaly of P1 and P2 segments of PCA remains to be studied. Since the occipital lobe of the cerebrum receives blood supply either from ICA or from vertebrobasilar artery, the clinical significance of hemodynamic changes due to morphologic and/or morphometric variations is likely to be higher for its clinical applications.

In the presence of the aplasia of P1 segment of PCA, blood supply to the occipital lobe would be solely dependent on intact ICA while in the presence of the aplasia of PCoA blood supply to the occipital lobe would be solely dependent on intact vertebrobasilar artery. The clinical importance of 2.35% of aplasia cannot be stated at this stage.

P1 segment is usually absent in early embryonic life, when C-R length of the embryo is between 5 mm and 40 mm. Since P1 segment develops only after gaining of C-R length of 40 mm in later stage of embryonic life, the blood supply to the occipital lobe is totally dependent on ICA. A small blood vessel originates from rostral end of the basilar artery and joins the branch from the internal carotid artery going to the occipital lobe to form the PCA. These developmental observations were reported by Padget in 1948 [12]. In the present study, we found 2 cases (1.17%) of true early embryonic type of PCA (Figure 8).

P2 segment is therefore classified as either adult or fetal as per the contribution from an ICA or from vertebrobasilar artery.

Separate studies on PCA are very few. Only the proximal portion of PCA associated with CW is studied in detail as is seen in the literature, whereas the distal portion of PCA-P2 and its morphological variations are described to a lesser extent.

Branching patterns of PCA are hardly found in the literature. The only people who studied the branching patterns of PCA in detail were Ladzinski and Maliszewski [3] and we have compared the branching pattern of PCA with them (Table 1). Complex branching pattern of PCA is simplified in the present study. The branching pattern of PCA is divided in 6 groups. Each group is further divided into types forming 16 overall possibilities. The most common branching pattern found in the present study was Group I-Type 1 (20.58%). In comparison with Ladzinski and Maliszewski [3], we found a lot of differences in incidence of branching pattern. Such differences can be attributed to racial variation. Studies on larger scale in the future all over the world shall provide more insights over the most prevalent branching pattern of PCA.

Amongst 6 groups, Group II was the most commonly seen in the present study (41.75%).

Group III, Group IV, and Group V were seen as the least found branching groups (0.29%) of the present study.

Pai et al. [7], 2007, have studied the microsurgical anatomy of the posterior circulation in 25 Indian cadaveric brains. As mentioned by them, not studying cortical branches of the PCA was the limitation of their study. The present study described the branching pattern of cortical branches.

The present study describes observations over symmetry of PCA. In addition, symmetry of P1 and P2 segments of PCA has also received special attention for the first time. An isolated study by Al-Hussain et al., 2001 [6], documented the asymmetrical PCA in 28%; however, their study did not specify symmetry of P1 and P2 segments of PCA separately. The present study observed asymmetry of 30% and 40% of P1 and P2 segments, respectively.

Morphometric observations of P1 segment of PCA of the present study in addition to the previous studies are summarized in Table 4. Results of the present study are comparable with the other research workers, while the present study has additionally documented the morphometry of P2 segment. Mean length and mean diameter of P2 segment found in our study were 52 mm and 2.7 mm, respectively, on both sides.

Although morphometric results of P1 segment are similar to Pai et al., results of P2 segments are different because they have divided the P2 segment of PCA into four subparts.

Pai et al. divided the posterior cerebral artery into four segments. The length of the P2 segment varied from 12 to 28 mm with the mean of 19.9 mm. The average length of the P3 segment varied from 13 to 38 mm with the mean of 22.4 mm. In the present study, the length of P2 varied from 35 to 75 mm with the mean of 52 mm. The length of P2 in the present study is more or less equivalent to the length of P2 plus P3 of Pai et al. [7].

The anomalous origin of PCA from ICA has a great significance in obstruction or embolism of internal carotid and common carotid arteries. Ligation of one of these arteries may interrupt the blood supply to the large area of the brain [13].

Thrombosis or embolism affecting the internal carotid may cause infarction of the occipital pole when the PCA originates from the ICA. On the contrary, this anatomical variation may prevent occipital pole infarction in basilar thrombosis. Though CW serves a stabilizing function, the possibility of inadequacy still exists for the occurrence of brain infarction in occlusion of carotid and vertebral artery. Functional failure of the CW could arise from anatomical anomalies or obstructive vascular disease [14].

The individual morphological variations of P1 and P2 segments of PCA from the present study provide valuable addition which is likely to be useful in vascular pathophysiology and treatment.

## 5. Conclusion

The present study provides the complete description of PCA regarding its morphology, branching pattern, symmetry, and morphometry. Awareness of these anatomical variations described shall prove to be useful for cerebrovascular procedures. Since anatomical studies of PCA with special reference to P2 segment and branching pattern are highly limited, similar studies all over the world on large scale would help establish the overall significance of P2 segment of PCA.

## Figures and Tables

**Table 1:** Branching pattern of PCA.

Groups	Schema number	Schema pattern	Ladzinski and Maliszewski [3] number of cases	Present study			
				Number of cases	Percentage	Total number of cases	Total percentage
I	1	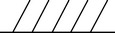	7	70	20.58	70	20.58

II	2	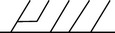	10	28	8.23	142	41.75
	3	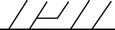	6	28	8.23		
	4	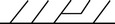	10	64	18.82		
	5	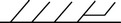		22	6.47		

III	6	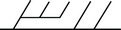	7	0	—	1	0.29
	7	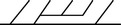	10	1	0.29		
	8	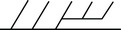	6	0	—		

IV	9	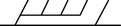	12	1	0.29	1	0.29
	10	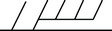		0	—		

V	11	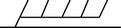		1	0.29	1	0.29

VI	12	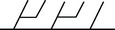	19	45	13.23	120	25.28
	13	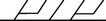	3	35	10.29		
	14	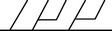		40	11.76		

VII	15	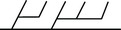	4	3	0.88	5	1.46
	16	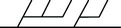		2	0.58		

**Table 2:** Morphometric observations (in millimeters).

Dimension	PCA segment	Right				Left			
		Min.	Max.	Mean	SD	Min.	Max.	Mean	SD
Length	P1	1	20	7.8	0.31	3	15	7.8	0.29
	P2	35	73	52.2	10.2	35	75	52.1	10.1

Diameter	P1	1	5	2.8	0.08	1	5	2.8	0.08
	P2	2	4	2.7	0.04	2	4	2.7	0.04

**Table 3:** Comparison of morphological variations of P1.

Variation type of P1	Present study %	Kapoor et al. [4]	Alpers et al. [5]	Al-Hussain et al. [6]
Absent	**2.35**	—	—	—
Hypoplasia	**5.29**	10.6	6.3	1
Duplication	**2.35**	2.4	—	—
Fenestration	**1.17**	—	—	—
SCA + P1 stem	**1.76**	—	—	—

**Table 4:** Comparison of morphometry of PCA.

PCA	Present study	Pai et al. [7]	Moore et al. [8]	Krishnamurthy et al. [9]	Krabbe-Hartkamp et al. [10]	Kamath [11]
Length	**7.5**	7.5	6.75	—	—	6.9
Diameter	**2.8**	2.7	1.7	2.13	2	2.2
